# GABA-Producing Natural Dairy Isolate From Artisanal Zlatar Cheese Attenuates Gut Inflammation and Strengthens Gut Epithelial Barrier *in vitro*

**DOI:** 10.3389/fmicb.2019.00527

**Published:** 2019-03-18

**Authors:** Svetlana Sokovic Bajic, Jelena Djokic, Miroslav Dinic, Katarina Veljovic, Natasa Golic, Sanja Mihajlovic, Maja Tolinacki

**Affiliations:** Laboratory for Molecular Microbiology, The Institute of Molecular Genetics and Genetic Engineering, University of Belgrade, Belgrade, Serbia

**Keywords:** GABA, lactobacilli, artisanal food, antimicrobial, anti-inflammatory activity

## Abstract

Probiotic bacteria are recognized for their health-promoting properties, including maintenance of gut epithelial integrity and host immune system homeostasis. Taking into account the beneficial health-promoting effects of GABA, the presence of the *gadB* gene, encoding glutamate decarboxylase that converts L-glutamate to GABA, was analyzed in Lactic Acid Bacteria (LAB) natural isolates from Zlatar cheese. The results revealed that 52% of tested *Lactobacillus* spp. and 8% of *Lactococcus* spp. isolates harbor the *gadB* gene. Qualitative and quantitative analysis of GABA production performed by thin-layer chromatography (TLC) and high-performance liquid chromatography (HPLC) revealed the highest GABA production by *Lactobacillus brevis* BGZLS10-17. Since high GABA-producing LAB natural isolates are the most valuable source of naturally produced GABA, the probiotic properties of BGZLS10-17 were characterized. This study demonstrated high adhesion of BGZLS10-17 strain to Caco-2 cells and the ability to decrease the adhesion of *Escherichia coli* ATCC25922 and *Salmonella enterica* C29039. Treatment of differentiated Caco-2 cells monolayer with BGZLS10-17 supernatant containing GABA alleviated inflammation (production of IL-8) caused by IL-1β and significantly stimulated the expression of tight junction proteins (zonulin, occludin, and claudin 4), as well as the expression of *TGF-β* cytokine leading to the conclusion that immunosuppression and strengthening the tight junctions can have significant role in the maintenance of intestinal epithelial barrier integrity. Taken together the results obtained in this study support the idea that using of GABA producing BGZLS10-17 probiotic strain could be a good strategy to modulate immunological response in various inflammatory diseases, and at the same time, it could be a good candidate for adjunct starter culture for production of GABA-enriched dairy foods and beverages offering new perspectives in designing the novel functional foods.

## Introduction

γ-amino butyric acid (GABA) is the major inhibitory neurotransmitter in the mammalian central nervous system, directly affecting the personality and the stress management, and has hypotensive, tranquilizing, diuretic and antidiabetic effects (Li and Cao, 2010; Dhakal et al., 2012). The protective role of GABA is demonstrated in many autoimmune diseases such as type 1 diabetes (Tian et al., 2004), experimental autoimmune encephalomyelitis (EAE), an animal model of multiple sclerosis (Bhat et al., 2010), collagen-induced arthritis (Kelley et al., 2008; Tian et al., 2011), and contact dermatitis (Nigam et al., 2010). This GABA effect is usually interpreted through the inhibitory effect of this molecule on the immune system. On the other hands, different human and experimental autoimmune models have been characterized by intestinal tight junction dysfunction (Visser et al., 2009). Disruption of intestinal homeostasis and increased intestinal permeability have been shown as an early and immune-mediated event in EAE (Nouri et al., 2014), inflammatory bowel disease (Suenaert et al., 2002), and celiac disease (Vogelsang et al., 1998). In addition, different infectious agents affect intestinal permeability in order to penetrate into deeper tissues (Wang et al., 2018), while entry of unwanted antigens, due to the leaky intestinal barrier, can lead to systemic inflammatory response syndrome, characterized by a whole body inflammatory state, and multiple organ failure (Liu et al., 2005). Thus, intestinal permeability represents a potential therapeutic target in treatment/prevention of MS and other autoimmune and inflammatory diseases.

Many drugs and bioactive components targeting GABA receptors are widely used in pharmaceutical and food industry. It is believed that GABA of extra brain origin, except through the pituitary gland, cannot pass the blood-brain barrier, although the results of studies dealing with this issue are inconsistent (Kakee et al., 2001; Powers et al., 2008; Boonstra et al., 2015). GABA contributes to gut-brain signaling through different pathways including enteric neurons, entero-endocrine cells and immune cells (Mazzoli et al., 2017). The microbial biosynthesis of GABA occurs in simple, highly efficient, and environmentally friendly reaction (Dhakal et al., 2012.). The biosynthesis of GABA occurs in a single step. Glutamate decarboxylase (GAD) (EC 4.1.1.15), a pyridoxal 5^′^-phosphate-dependent enzyme, catalyzes the irreversible α-decarboxylation of L-glutamate to GABA (Ueno, 2000).

Lactobacilli known for their beneficial effects on human and animal health, represent the most important group of GABA producers (Cho et al., 2007; Lebeer et al., 2008). GABA production was detected in *Lactobacillus brevis*, *Lb. paracasei*, *Lb. delbrueckii* subsp. *bulgaricus*, *Lb. buchneri*, *Lb. plantarum*, *Lb. helveticus*, and *Lb. rhamnosus*, mostly isolated from traditional fermented foods such as cheese, kimchi and sour dough (Cho et al., 2007; Siragusa et al., 2007; Komatsuzaki et al., 2008; Seok et al., 2008; Kim et al., 2009; Sun et al., 2009). Our previous results showed considerable diversity among LAB natural isolates from artisanal dairy products in Western Balkan with great technological and probiotic potential (Golić et al., 2013; Uroić et al., 2014). Due to increased public awareness of the use of natural food compounds, higher attention has been made on the use of metabolites produced by LAB isolates from artisanal products. In this study, natural isolates originating from Zlatar cheese were used. Zlatar cheese is an artisanal cheese manufactured in the remote households on the highlands of the nature reserve, mountain Zlatar, Serbia. Natural dairy LAB isolates from Zlatar cheese produce antimicrobial compounds with broad inhibitory spectrum, exopolysaccharides (EPS) with specific immunomodulatory activity and various other bioactive compounds with bile salt hydrolase activity and ability of cholesterol assimilation (Veljovic et al., 2007).

Although the immunomodulatory activity of GABA on different immune cells have been repeatedly proven, there is no references about the effects of GABA on enterocytes and intestinal integrity in inflammatory condition. Just a few references investigated the effect of GABA on enterocytes and intestinal integrity in different condition. Braun et al. (2015) proposed that the positive effects of glutamine on intestinal integrity are partly attributable to the promoting effects of its metabolite GABA on the expression of protective mucin in enterocytes. El-Hady et al. (2017) showed that administrated GABA have a role in improving histopathological and biochemical disturbances in the rat’s small intestine following gamma radiation. Considering protective effects of GABA on stressed enterocytes showed in these studies as well as repeatedly proven positive effect of different *Lactobacillus* strains, we aimed to investigate the effects of GABA-producing *Lactobacillus* strain on enterocytes exposed to inflammatory condition *in vitro*. Additionally, the other health-promoting effects of GABA-producing LAB natural isolates from Zlatar cheese was investigated in different *in vitro* settings.

## Materials and Methods

### Bacterial Strains, Media and Growth Conditions

Bacterial strains used in this study are presented in Table 1. The *Lactobacillus* strains were grown in De Man-Rogosa-Sharpe (MRS) medium (Merck GmbH, Darmstadt, Germany), while *Lactococcus* strains were grown in M17 medium (pH 7.2) (Merck GmbH) supplemented with 0.5% (w/v) glucose (GM17). Bacteria were grown at 30 or 37°C, under aerobic or anaerobic conditions, depending on the strain. Anaerobic conditions were achieved by using the Anaerocult A (Merck GmbH) in anaerobic jars. Solid medium was prepared by adding agar (1.5%, w/ v) (Torlak, Belgrade, Serbia) to the broth medium. The strains were maintained at -80°C in an appropriate medium (GM17 or MRS) supplemented with 15% (v/v) glycerol.

**Table 1:** The list of strains used in this study.

Species	*gadB*-negative strains	*gadB*-positive strains	GABA producing strains
*Lactobacillus brevis*	BGZLS30-23	BGZLS45-36	BGLMM10, BGLMM11, BGZLS10-17, BGZLS30-2
*Lactobacillus paracasei* subsp. *paracasei*	BGZLS10-6, BGZLS45-50, BGZLS60-32	BGZLS10-1, BGZLS20-1, BGZLS45-25, BGZLS60-50	BGZLS45-49
*Lactobacillus paraplantarum*	BGZLS60-58		
*Lactobacillus plantarum*	BGZLS60-59	BGZLS60-43	BGZLS20-20, BGZLS30-41
*Lactobacillus rhamnosus*	BGZLS10-2		
*Lactococcus lactis*		BGZLS10-33	
*Lactococcus lactis* subsp. *lactis*	BGZLS1-11, BGZLS10-35	BGZLS10-34	


### DNA Manipulation and Identification of GABA-Producing Strains

The total DNA of lactobacilli strains was extracted using the QIA DNA Mini Kit (Qiagen GmbH, Hilden, Germany). The PCR amplification of 16S rDNA, with UNI16SF (5^′^-GAGAGTTTGATCCTGGC-3^′^) and UNI16SR (5^′^-AGG AGGTGATCCAGCCG-3^′^) primers was performed as described by Jovčić et al. (2009). To amplify highly conserved region of *gad* gene, CoreF (5^′^-CCTCGAGAAGCCGATCGCTTAGTTCG-3^′^) and CoreR (5^′^-TCATATTGACCGGTATAAGTGATGCCC-3^′^) primers were used as described by Siragusa et al. (2007). Briefly, 25 μl reaction contained 500 ng/μl of DNA, 0.5 U of Q5 High-Fidelity DNA Polymerase (New England Biolabs, Inc., Ipswich, MA, United States), 200 μM of each deoxynucleoside triphosphate, 0.5 μM of both primers and 1x Q5 Reaction Buffer. The obtained PCR amplicons were purified (Qiagen) and sequenced (Macrogen, Amsterdam, the Netherlands). Sequence annotation and the database searches for sequence similarities were performed with the BLAST tool available online^1^.

### Analysis of GABA Production

The strains were incubated in MRS or GM17 medium supplemented with various concentrations of monosodium glutamate-MSG (Acros organics, Morris Plains, NJ, United States) (0.5–4%) for 48 h. The cells were harvested by centrifugation (4500 × *g* for 15 min at 4°C) and 1 ml of the supernatant was evaporated up to 200 μl and diluted 2-fold by 7% acetic acid. The samples were centrifuged at 8100 ×*g* for 15 min at room temperature. Obtained supernatants were used for thin-layer chromatography (TLC) analysis (Park et al., 2014.) and high-performance liquid chromatography (HPLC) (Holdiness, 1983). Briefly, 1 μl of supernatant was spotted on a silica gel 60F_254_ TLC plate (Macherey-Nagel, Düren, Germany) and developed with *n*-butanol, acetic acid, and water (4:1:1 v/v/v). Upon development was complete the plate was dried and visualized with 0.2% ninhydrin reagent (Sigma, Chemical Co., St. Louis, MO, United States). The conversion rate of MSG to GABA was analyzed using ImageJ software. The aliquots of 100 μl [bacterial supernatants and GABA standard (Sigma)] were filtrated through 0.22 μm filters and derivatized to phenylthiocarbamyl-GABA (Rossetti and Lombard, 1996). The derivatized samples were dissolved in 200 μl of initial mobile phase, solution A (138 mM sodium acetate, pH 6.3, 6% acetonitrile, 0.05% triethylamine). HPLC separation was performed on the instrument of Thermo scientific 3000 equipped with a Hypersil gold column (Thermo Fisher Scientific, Waltham, MA, United States 150 × 4.6, 5 μm). The elution solvent system comprised of solution A, solution B (acetonitrile) and solution C (water). The elution program is shown in Table 2. The amount of GABA production was calculated from GABA standard curve.

**Table 2:** Elution program of HPLC.

Time (min)	Solution A (%)	Solution B (%)	Solution C (%)	Flow (ml/min)
0	60	12	28	0.6
6	60	12	28	0.6
6.1	20	13.5	66.5	0.4
22	20	13.5	66.5	0.4
25	60	12	28	0.6
40	60	12	28	0.6


### The Cumulative Effect of Simulated Gastrointestinal Transit on Survival of Selected Strains

The survival of LAB strains during the passage through gastrointestinal tract (GIT) was monitored in an *in vitro* model that simulates the physiological conditions as described by Sánchez et al. (2010). *Lactobacillus* isolates from 10 ml of overnight cultures in MRS medium were harvested by centrifugation (4500 ×*g,* 10 min), washed in 0.85% NaCl and resuspended in gastric, duodenal and intestinal juice(s) (Sánchez et al., 2010) at pH 2.0 and pH 8.0 supplemented with 10% reconstituted skimmed milk. The aliquots were taken at 0, 90, and 180 min and serial 10× dilutions in 0.85% NaCl were plated on MRS agar plates. The plates were incubated anaerobically at 37°C for 48 h. The results were expressed as Log colony forming units (cfu) ml^-1^ and the survival rate was calculated from the viable cell count with respect to initial cell counts. The experiments were performed in triplicates.

### Gastric Tolerance at pH 2.0 in the Presence of Skim Milk, Milk Proteins, and Mucin

One mg ml^-1^ of milk powder (Mlekara d.o.o., Pancevo, Serbia), β-lactoglobulin, and mucin (Sigma) were resuspended in gastric juice at pH 2.0 (125 mM NaCl, 7 mM KCl, 45 mM NaHCO_3_ and 0.3% pepsin). Skim milk was prepared according to the manufacturer’s instructions at 110 mg ml^-1^. Lactobacilli strains from an MRS overnight culture were harvested by centrifugation (4500 ×*g*, 10 min), washed twice in 0.85% NaCl (Merck) and resuspended in 10 ml of an appropriate gastric juice containing milk proteins, β-lactoglobulin, skim milk or mucin. The mixtures were incubated anaerobically for 2 h at 37°C. The tolerance of the tested strains was calculated as described in previous section.

### Antibiotic Susceptibility Testing

Determination of the minimal inhibitory concentration (MIC) was performed by microdilution tests in Iso-Sensitest Broth (Oxoid, Hampshire, United Kingdom). The MIC breakpoints of eight antibiotics (ampicillin, gentamicin, kanamycin, streptomycin, erythromycin, clindamycin, tetracycline, and chloramphenicol) were determined in accordance to European Food Safety Authority recommendations (EFSA, 2008). Appropriate cell culture was added in wells of the microtiter plate containing increasing concentrations of antibiotics dissolved in 180 μL ISO medium. Cell density was monitored by OD_600_ measurements after 24 h of incubation at 37°C in a microtiter plate reader (Tecan Austria GmbH, Grödig, Austria). The lowest concentration of antibiotic at which no growth of bacteria was detected was taken as MIC. Experiments were done in triplicate.

### Hemolytic Activity

*Lactobacillus brevis* strains were cultured in MRS broth and were streaked on Columbia agar plates containing 5% of sheep blood (Oxoid). The plates were incubated for 48 h at 30°C. According to Argyri et al. (2013), blood agar plates were examined for signs of β-haemolysis (clear zones around colonies), α-haemolysis (green-hued zones around colonies) or γ-haemolysis (no zones around colonies).

### Gelatinase Activity

The phenotypic assay was performed as described by Su et al. (1991). Briefly, the lactobacilli strains were grown on agar plates containing 3% gelatine (Oxoid) at 37°C for 48 h and flooded with a saturated solution of ammonium sulfate (Centohem, Stara Pazova, Serbia). A transparent halo around cells and gelatine precipitates indicated gelatinase producers. As a positive control *E. faecalis* V583 (Paulsen et al., 2003) was used.

### Antimicrobial Activity

All *Lb. brevis* strains and their supernatants were tested in triplicates for their antimicrobial activity against following pathogen strains: *Bacillus cereus* ATCC 11778, *Bacillus spizizeni* ATCC 6633, *Citrobacter freundii* ATCC 43864, *Enterococcus faecalis* ATCC 29212, *Escherichia coli* ATCC 25922, *Listeria innocua* ATCC 33090, *Listeria ivanovii* ATCC 19119, *Listeria monocytogenes* ATCC 19111, *Proteus hauseri* ATCC 13315, *Proteus mirabilis* ATCC 12453, *Pseudomonas aeurginosa* PAOI, *Rhodococcus equi* ATCC 6936, *Salmonella enterica* C2 9039, *Salmonella typhimurium* ATCC 14028, *Shigela sonnei* ATCC 29930, *Staphylococcus aureus* ATCC 25923, *Staphylococcus epidermidis* ATCC 12228, and *Yersinia enterocolitica* ATCC 27729. For production of antimicrobial compounds, *Lb. brevis* strains were inoculated (1%, v/v) in MRS broth and incubated for 16 h. Both, the overnight cultures and the supernatants obtained after centrifugation (12,000 ×*g*, 5 min) were tested for antimicrobial activity by the agar-well diffusion assay against indicator pathogen strains. Soft LB and BHI soft agar (0.7% w/v) containing pathogenic strains, was overlaid onto LB and BHI agar plates, respectively. Wells were made in the lawn of hardened soft agars. Aliquots (50 μl) of supernatants and overnight cultures were poured into the wells. A clear zone of inhibition around the well was taken as a positive signal for antimicrobial activity.

### Adherence to Caco-2 Cells

The colonocyte-like cell lines Caco-2, was used to determine the adhesion ability of bacterial strains. Caco-2 was purchased from the European Collection of Cell Cultures (ECACC No. 86010202). The culture and maintenance of the cell line was carried out following standard procedures (Sánchez et al., 2010) using Dulbecco’s Modified Eagle Medium (DMEM) supplemented with 10% fetal bovine serum (FBS), 100 U ml^-1^ penicillin and 100 mg ml^-1^ streptomycin and 2 mM l-glutamine. Media and reagents were purchased from Thermo Fisher Scientific. Caco-2 cells were seeded in 24-well plates and cultivated until monolayers formed with no further visible differentiation. For adhesion experiments, 13 ± 1 day-old cellular monolayers were used. Overnight bacterial cultures (24 h) were washed twice with PBS solution. The pellets were resuspended in the corresponding cell-line media without antibiotics at a concentration of about 10^8^ cfu ml^-1^. Cellular monolayers were also carefully washed and bacterial suspensions were added at a ratio of 10:1 (bacteria : eukaryotic cell). Following co-incubation for 1 h at 37°C and 5% CO_2_ the cells were gently washed and lysed with 0.25% Trypsin–EDTA solution (Sigma). Serial dilutions of samples, before and after adhesion, were diluted in PBS and plated on MRS-agar plates. The adherence (expressed as a percentage) was calculated as: cfu adhered bacteria/cfu added bacteria. Experiments were performed in two replicated plates and in each plate three wells were used per sample.

### Competitive Exclusion Assay

Exclusion of pathogen strains *Escherichia coli* ATCC25922 and *Salmonella enterica* C29039 was done to Caco-2 cell line by *Lactobacillus brevis* strains as described previously (Živkovic et al., 2016). Briefly, bacterial cultures were washed twice with PBS and resuspended in DMEM without antibiotics at a concentration of ∼1 × 10^7^ CFU ml^-1^. The bacterial suspensions containing pathogen or a combination of pathogen strain and lactobacilli (ratio 1:1) were independently added to the Caco-2 monolayers at a ratio of 10:1 (bacteria : eukaryotic cells) and incubated at 37°C, with 5% CO2 for 1 h. Afterward, the monolayers were gently washed twice with PBS and lysed with 0.25% Trypsin–EDTA solution (Sigma). Associated *E. coli* and *S. enterica* strains were counted by plating the serial 10-fold dilutions of the suspension on LA plates. The percentage of *E. coli* and *S. enterica* strains association was calculated as follows: 100 × CFU ml^-1^ bacteria associated/CFU ml^-1^ bacteria added. Each combination was tested in triplicate. To determine the capability of the lactobacilli to decrease the association of pathogen strains to Caco-2 monolayers, data were referred to that obtained with the *E. coli* and *S. enterica* strains alone, respectively (i.e., 100% association).

### Epithelial Integrity Analysis, Inflammation Induction and Treatments

*Lactobacillus brevis* BGZLS10-17, the best GABA producing strain, was used to determine the expression of tight junction proteins (zonulin, occludin, and claudin 4) and cytokine production (IL-8 and TGF-β) in differentiated Caco-2 cells. Caco-2 cells were seeded in 24 well plate and cultivated until monolayers formed with further differentiation for 21 days. Cell culture medium was changed every second day during the 21-day differentiation period.

Bacterial culture was grown in MRS medium and according to HPLC analysis there was no GABA production. To stimulate GABA production by BGZLS10-17, MRS medium was supplemented with 0.6% of MSG (Acros organics). Differentiated Caco-2 cells were treated with BGZLS10-17 supernatants in concentrations of 0.625 %, 1.25 % and 2.5 %, with 1 mM, 2 mM and 4 mM GABA respectively, determined by HPLC. Supernatant from the bacterial culture grown in MRS (the final concentration of 2.5%) was used as control. This supernatant with addition of artificial GABA (Sigma) in final concentration of 4 mM was used as an additional control in order to correlate the effect of supernatant with GABA produced by bacteria. Supernatant was obtained from 48 h bacterial culture and neutralized to pH about 7. Additionally, cells were treated with recombinant human IL-1β beta (R&D systems, Minneapolis, MN, United States), as a trigger of inflammation, and it was monitored whether a supernatant containing 4 mM GABA can alleviate the induced inflammation. All treatment lasted 24 h.

### Cytotoxicity Assay

The level of cytotoxicity in the cell cultures was measured by lactate dehydrogenase (LDH) Cytotoxicity Assay Kit (Thermo Fisher Scientific) which detects LDH released from dead cells. After treatments, supernatants were collected and LDH activity was determined by following the manufacturer’s instructions. The absorbance was measured at 450 nm on a microplate reader (Tecan).

### Quantitative Real-Time PCR

Total RNA was extracted from Caco-2 as previously described by Lukic et al. (2013) with slight modifications. Cells were lysed in denaturing solution (4 M guanidine thiocyanate, 25 mM sodium citrate, 0.1 M *b*-mercaptoethanol, 0.5% [wt/vol] *N*-lauroylsarcosinate sodium salt) followed by acid phenol (pH 4) extractions and isopropanol precipitation. cDNA was generated from 0.5 μg total RNA according to the reverse transcriptase manufacturer’s protocol (Thermo Fisher Scientific). Quantitative PCR was carried out on 7500 real-time PCR system (Applied Biosystems, Waltham, MA, United States) using KAPA SYBR Fast qPCR Kit (Kapa Biosystems, Wilmington, MA, United States) under the following conditions: 3 min at 95°C activation, 40 cycles of 15 s at 95°C and 60 s at 60°C. For β-actin cDNA amplification were used β-actin forward (5^′^TTGCTGACAGGATGCAGAAGGAGA3^′^), and reverse (5^′^TCAGTAACAGTCCGCCTAGAAGCA3^′^) (Li et al., 2015). Following primers were used for the claudin cDNAamplification CLDN 4 forward (5^′^ACAGACAAGCCTTACTCC3^′^) and reverse (5^′^GGAAGAACAAAGCAGAG3^′^), occludin cDNA amplification OCLN forward (5^′^TCAGGGAATATCCACCTATCACTTCAG3^′^) and reverse (5^′^CATCAGCAGCAGCCATGTACTCTTCAC3^′^) and for zonulincDNA amplification ZO-1 forward (5^′^AGGGGCAGTGGTGGTTTTCTGTTCTTTC3^′^) and reverse (5^′^GCAGAGGTCAAAGTTCAAGGCTCAAGAGG3^′^) (Elamin et al., 2012). For expression of IL-8 were used cIL-8 forward (5^′^GGCACAAACTTTCAGAGACAG3^′^) and reverse (5^′^ACACAGAGCTGCAGAAATCAGG3^′^) primers (Angrisano et al., 2010). For the TGF-β cDNA expression were used TGF-β_F forward (5^′^AAGGACCTCGGCTGGAAGTGG3^′^) and reverse (5^′^CCGGGTTATGCTGGTTGTACAG3^′^) (Dragicevic et al., 2017). Expression of the tight junction proteins and cytokines mRNAs was normalized against β-actin mRNA expression. All used primers were purchased from Thermo Fisher Scientific.

### Western Blot

Proteins were isolated from Caco-2 cells using RIPA buffer and subsequently subjected to Western blot analysis as described by Dinić et al. (2017). Briefly, the extracted proteins (10 μg) were separated on 12% SDS–PAGE and transferred to 0.2 mm nitrocellulose membrane (GE Healthcare, Chicago, IL, United States) using Bio-Rad Mini trans-blot system (Bio-Rad, Hercules, CA, United States). The membranes were incubated for 2 h with anti-claudin (CLDN-4) antibody (1:1000; Novus Biologicals, United States) and anti-β-actin (1:1000; Thermo Fisher Scientific). The membranes were washed and incubated with appropriate HPR-conjugated secondary antibodies (goat anti-rabbit; 1:10000; Thermo Fisher Scientific) for 1 h at room temperature. Proteins were detected by enhanced chemiluminescence (Immobilon Western, Merck Millipore).

### Statistical Analysis

All data are presented as mean values ± standard error (SD). One-way ANOVA with the Tukey’s *post hoc* test was used to compare multiple groups. Values at *p* < 0.05 or less were considered statistically significant. All experiments were repeated at least three times. Statistical analysis was carried out using SPSS 20.0 for Windows.

## Results and Discussion

“Probiotics are live microorganisms that confer health benefits to the host when ingested in adequate amounts” (WHO-FAO, 2006). Various mechanisms staying behind the health-promoting activity of probiotic bacteria have been proposed, including antimicrobial activity, positive influence on gut microbiota composition, competitive adhesion to gut mucosa and intestinal epithelial cells (IEC), modulation of the immune response, strengthening the gut epithelial barrier and producing of various important bioactive molecules, including vitamins, amino acids and GABA, among others (Bermudez-Brito et al., 2012). The aim of this study was the characterization of GABA-producing natural LAB isolates in order to be eventually used in formulation of added-value fermented foods with GABA.

### Evaluation of GABA Producing LAB Strains

**FIGURE 1:**
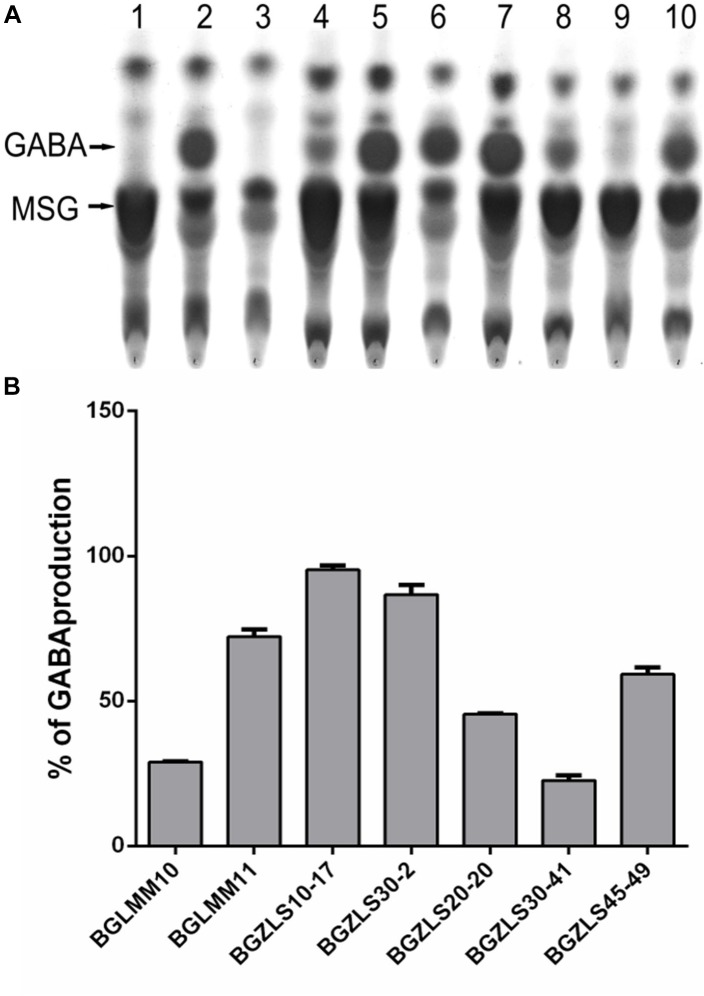
**(A)** TLC chromatogram of GABA production. Lane 1, MSG in MRS; Lane 2, GABA in MRS; Lane 3. MRS; Lane 4, BGLMM10; Lane 5, BGLMM11; Lane 6, BGZLS10-17; Lane 7, BGZLS30-2; Lane 8, BGZLS20-20; Lane 9, BGZLS30-41; Lane 10, BGZLS45-49. Arrows indicate MSG and GABA. **(B)** Conversion of MSG to GABA. The percent of MSG conversion to GABA is indicated on *y*-axis. The bars represent GABA production of *Lactobacillus* isolates. Data from three independent experiments are expressed as mean ± SD.

In order to select natural LAB isolates with the highest GAD activity, LAB strains previously isolated from Zlatar cheese (Veljovic et al., 2007) were screened for presence of the *gadB* gene by PCR amplification. Out of 25 LAB isolates from Zlatar cheese 15 strains (60%), including 13 lactobacilli (52%) and two lactococci (8%) were positive for presence of the *gadB* gene (Table 1). The production of GABA was determined by TLC analysis in seven out of 15 analyzed lactobacilli strains (28% in total from 25 LAB strains) (Figure 1A). In comparison, Siragusa et al. (2007) revealed 61 GABA-producing isolates out of 440 randomly taken gram-positive, catalase-negative, non-motile, and acidifying isolates (13.86%) from various Italian cheeses, while Franciosi et al. (2015) determined 68 out of the 97 GABA-producing isolates (70.10%) from alpine raw milk “*Nostrano*-cheeses.”

### Survival of GABA Producers in Simulated Gastrointestinal Conditions

**FIGURE 2:**
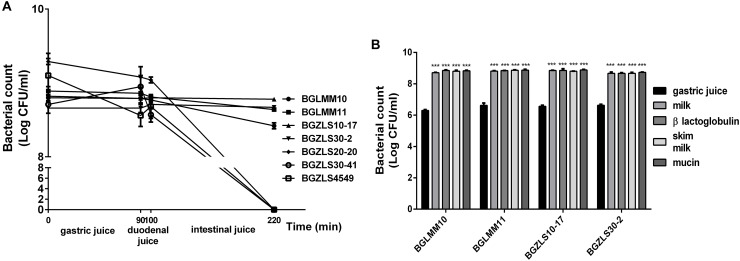
**(A)** Resistance to simulated gastrointestinal conditions. Survival of selected *Lactobacillus* isolates, as indicated on the right, under gastric conditions (0–90 min), duodenal (90–100 min) and intestinal conditions (100–220 min). The values are the averages of three replicates, and standard deviations are indicated by vertical bars. **(B)** Gastric tolerance at pH 2.0 in the presence of skim milk, milk proteins and mucin. All values are reported as a mean ± SD of 3 replicates. Statistical significance of gastric tolerance enhancement is shown (^∗∗∗^*p* < 0.001).

According to WHO-FAO (2006) the survival in simulated gastrointestinal (GI) conditions is an important criterion for selection of probiotic strains. It is particularly important for GABA-producing strains since they could synthesize GABA only if remain viable through GI passage. Survival of seven strains that effectively produce GABA was tested in simulated conditions of the gastrointestinal tract. Our results revealed that among seven GABA-producers only *Lb. brevis* strains BGLMM10, BGLMM11, BGZLS10-17 and BGZLS30-2 successfully survived after 220 min of incubation in chemically simulated GI transit (8.7–8.9 log CFU ml^-1^), when applied in one of the protecting carriers such as milk, skim milk, β-lactoglobulin, or mucin (Figure 2A,B). All tested carriers showed significant (*p* < 0.001; about two log cycles) improvement of survival, compared to survival of the strains applied in gastric juice (Figure 2B). The relatively high survival degree in the presence of bile salts in simulated small intestinal juice indicate resistance of some chosen strains to bile salts which could be ascribed to bile salts hydrolase (BSH) activity. BSH catalyzes the deconjugation of bile salts. Free, deconjugated bile salts have lower solubility at low pH because deconjugation increases their pKa values and precipitate as result of the fermentative metabolism of LAB (Begley et al., 2006).

### The Safety Status of the *Lb. brevis* GABA-Producing Strains

The absence of virulence factors (e.g., hemolytic and/or gelatinase activity) as well as acquired or transmissible antibiotic resistance is a safety prerequisite for selection of potential probiotic strains (WHO-FAO, 2002). The results revealed that none of the strains exhibited hemolytic and/or gelatinase activity (data not shown). Besides, in line with EFSA recommendations (EFSA, 2008) the susceptibility of the isolates to different antibiotics groups was evaluated. Cell wall inhibitors (ampicillin) and protein synthesis inhibitors (gentamicin, kanamycin, streptomycin, erythromycin, chloramphenicol, clindamycin, and tetracycline) were tested in order to confirm that the strains do not contain transferable resistance genes. The results showed that only the strain *Lb. brevis* BGZLS10-17 was susceptible to all tested antibiotics, while the strain *Lb. brevis* BGLMM11 was resistant to kanamycin (64 μgml^-1^), BGLMM10 to kanamycin (64 μgml^-1^) and tetracycline (16 μgml^-1^), and the strain and BGZLS30-2 to ampicillin (4 μgml^-1^), kanamycin (64 μgml^-1^) and tetracycline (16 μgml^-1^) (Table 3). Nevertheless, according to previous studies it is also important to differentiate the transferable resistance from natural intrinsic non-transmissible resistance (Danielsen and Wind, 2003; Argyri et al., 2013). The resistance to kanamycin has been confirmed for most *Lactobacillus* species and is considered intrinsic among lactobacilli due to the absence of cytochrome-mediated electron transport that mediates drug uptake (Temmerman et al., 2003; Argyri et al., 2013). The resistance to kanamycin and tetracycline was shown in *Lactobacillus paracasei* subsp. *paracasei* (Argyri et al., 2013). On the other hand, the results of Zhou et al. (2005) showed that *Lactobacillus* strains were susceptible to β-lactam antibiotics (penicillin, ampicillin, and cephalothin), but the frequency of transmission among LAB seems to be low because statistically only few LAB are beta lactam resistant. Therefore, the antibiotic resistances detected in *Lactobacillus* strains, could be considered intrinsic or natural resistances and, hence, non-transmissible.

**Table 3:** List of the tested antibiotics and MICs of four selected *L. brevis* isolates.

	Ampicillin	Vancomycin	Gentamicin	Kanamycin	Streptomycin	Erythromycin	Clindamycin	Tetracycline	Chloramphenicol
BGLMM10	1^S^	n. r	8^S^	64^R^	64^S^	0.5^S^	0.5^S^	16^R^	2^S^
BGLMM11	1^S^	n. r.	8^S^	64^R^	64^S^	0.5^S^	0.5^S^	8^S^	2^S^
BGZLS10-17	1^S^	n. r.	8^S^	32^S^	64^S^	0.5^S^	0.5^S^	4^S^	2^S^
BGZLS30-2	4^R^	n. r.	8^S^	64^R^	64^S^	0.5^S^	0.5^S^	16^R^	4^S^


*^S^, susceptible; ^R^, resistant; n. r., not required*.

### Quantification of GABA Production

The GABA produced by the strains *Lb. brevis* BGLMM10, BGLMM11, BGZLS10-17 and BGZLS30-20 was quantified by HPLC analysis. It appeared that the isolate *Lb. brevis* BGZLS10-17 produces the highest amount of GABA among all tested strains (6.4 ± 0.2 mgml^-1^ [62 ± 1.94 mM] at 1% MSG; the conversion rate of MSG to GABA was 95%) (Figure 1B). Interestingly, the strain *Lb. paracasei* NFRI 7415, isolated from traditional fermented crucians (tuna-sushi) in Japan, produced similar amount (60 mM) but after 144 h (6 days) cultivation (Komatsuzaki et al., 2005). The isolates *Lb. brevis* BGZLS30-2, BGLMM11 and BGLMM10 produced 5.8 ± 0.8 mgml^-1^ [56.2 ± 7.76 mM], 5.6 ± 0.6 mgml^-1^ [54.3 ± 5.82 mM], and 3.7 ± 0.2 mgml^-1^ [35.88 ± 1.94 mM] in MRS containing 1% MSG, respectively. The conversion rate of MSG to GABA was the lowest (30%) by the isolate *Lb. brevis* BGLMM10 while *Lb. brevis* BGZLS30-2 and BGLMM11 converted 86 and 72% of MSG to GABA, respectively (Figure 1B). On the other hand, various authors revealed that the GABA production could be enhanced by optimizing the culture conditions. For example, the strain *Lb. buchneri* MS, isolated from kimchi, produced GABA at a concentration of 251 mM with a 94% GABA conversion rate, under optimized conditions (Cho et al., 2007), while *Lactobacillus brevis* NCL912 in the optimized fermentation medium produced GABA in concentration of 345.83 mM (Li et al., 2010). Interestingly, although *Lc. lactis* NCDO218 produces more GABA with the higher glutamate supplementation, addition of arginine to the cell culture medium even more improves GABA production. Arginin stimulates glutamate decarboxylation, and the highest GABA production (8.6 mM) was observed when cell culture medium was supplemented together with glutamate and arginin (Laroute et al., 2016). Hence, the aim of our future work will be to optimize the cultivation conditions in order to further increase the GABA yield by the tested isolates.

### Antimicrobial Activity

**FIGURE 3:**
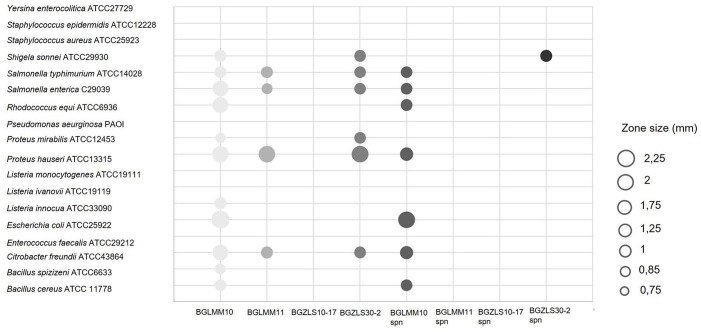
Antimicrobial activity of four *Lb. brevis* strains and their supernatants against clinically relevant pathogens. The zone of inhibition is represented by circles whereas the diameter of circle (mm) represents the size of inhibition.

Antimicrobial activity has been highly appreciated as a key property for selection of probiotic LAB as an alternative to antibiotics to fight against clinical pathogens (O’Shea et al., 2012). The antimicrobial activity of *Lb. brevis* strains against 18 pathogenic strains was determined (Figure 3). The *Lb. brevis* BGLM10, BGLM11, and BGZLS30-2 strains showed various degrees of antagonistic effects against number of clinically relevant pathogens (Figure 3). Interestingly, supernatant of the strain *Lb. brevis* BGLMM10 showed antagonistic effect against seven of 18 bacterial strains (*Salmonella typhimurium* ATCC14028, *S. enterica* C29039, *Rhodococcus equi* ATCC6936, *Proteus hauseri* ATCC13315, *Escherichia coli* ATCC25922, *Citrobacter freundii* ATCC43864, *Bacillus cereus* ATCC11778), while supernatant of the strain BGZLS30-2 showed antimicrobial activity against *Shigella sonnei* ATCC 29930. The negative results obtained by using pronase E revealed that antimicrobial activity is not of proteinaceous nature. The strain *Lb. brevis* BGZLS10-17 did not exhibit antimicrobial activity.

### Adhesion to Caco-2 Intestinal Epithelial Cells

**FIGURE 4:**
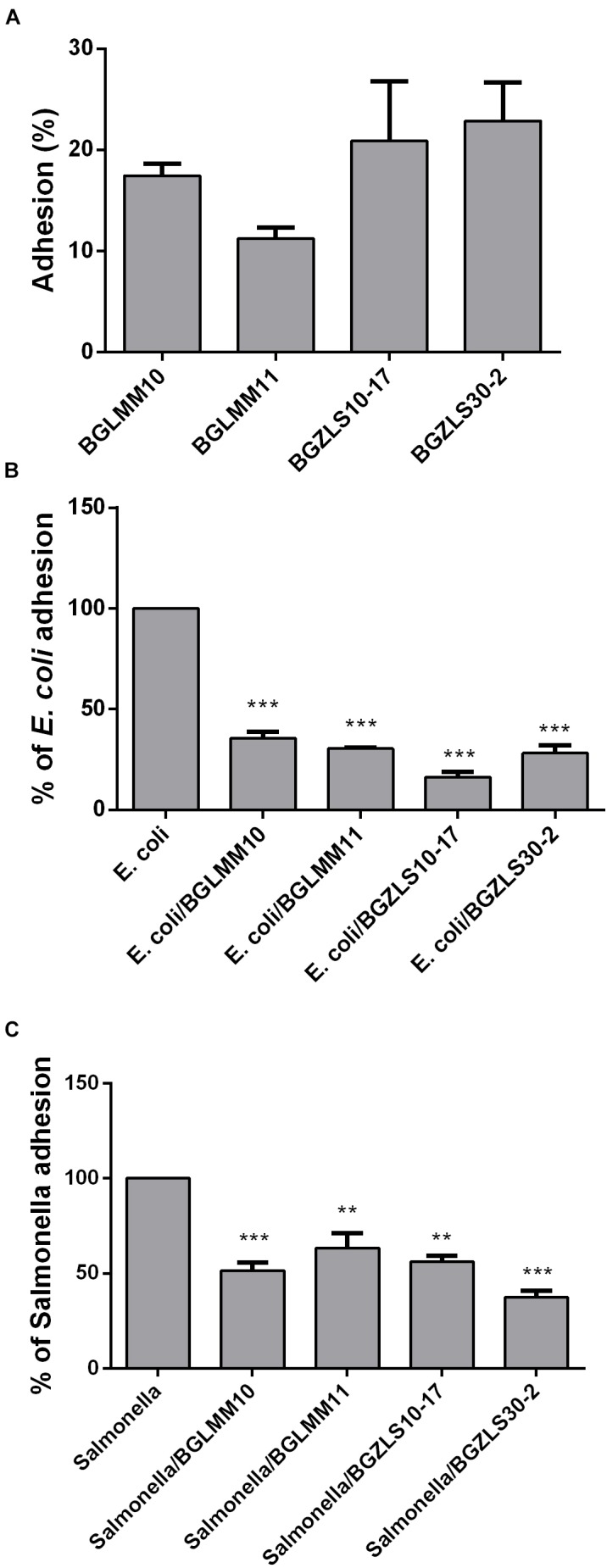
Adhesion of *Lb. brevis* BGLMM10, BGLMM11, BGZLS10-17 and BGZLS30-2 on Caco-2 cells **(A)** and association of *E. coli* ATCC25922 **(B)** and *Salmonella enterica* C29039 **(C)** to Caco-2 cells in the presence of *Lb. brevis* BGLMM10, BGLMM11, BGZLS10-17, and BGZLS30-2 strains; the adhesion of *E. coli* ATCC25922 and *S. enterica* C29039 in the absence of lactobacilli strains was inferred as 100%. Statistical significance of adhesion is shown (^∗∗^*p* < 0.01; ^∗∗∗^*p* < 0.001).

The ability of probiotic strains to adhere to IEC is an important criterion proposed by FAO/WHO guidelines for the selection of probiotic strains (WHO-FAO, 2006). Adhesion ability allows the strain to colonize intestinal mucosa and to persist in the intestine (Muñoz-Provencio et al., 2009). This is particularly important for the GABA-producers, since the maximal GABA production in these strains occurs after 48 h, hence this feature might be partly dependent on the persistence of the strain in the intestine and adhesion to mucosal surfaces. All four *Lb. brevis* strains were able to adhere to Caco-2 cells, although the adhesion varied among the strains (Figure 4A). The strain BGZLS30-2 exhibited the highest adhesion (22%), while the strain BGLMM11 showed the lowest adhesion to Caco-2 (11%) (Figure 4A).

In addition, the adhesive properties of probiotic strains are important health promoting property in term of their capability to competitively counteract the negative effects of pathogenic bacteria (WHO-FAO, 2006). This characteristic is shown to be strain dependent and attributed to the various cell surface components such as cell surface associated proteins (Varma et al., 2010), proteinaceous S-layer macromolecules (Zhang et al., 2010) aggregation factors (Miljkovic et al., 2015), as well as EPS (Zivkovic et al., 2015; Živkovic et al., 2016). The capability of the *Lb. brevis* strains to decrease the adhesion of *E. coli* ATCC25922 and *S. enterica* C29039 to the intestinal epithelium in the presence and absence of lactobacilli was tested. The results demonstrated high reduction of *E. coli* ATCC25922 and *S. enterica* C29039 adhesion in the presence of four tested *Lb. brevis* isolates (Figure 4B,C). The reduction of *E. coli* ATCC25922 adhesion to Caco-2 was 64, 70, 80 and 72%, while the reduction of *S. enterica* C29039 adhesion was 50, 37, 44, and 63% in the presence of BGLMM10, BGLMM11, BGZLS10-17 and BGZLS30-2 strains, respectively (the adhesion of *E. coli* ATCC25922 and *S. enterica* C29039 in the absence of lactobacilli strains was inferred as 100%) (Figure 4B,C).

### Effect of *Lactobacillus brevis* BGZLS10-17 on the Expression of Tight Junction Proteins and Cytokine Production

**FIGURE 5:**
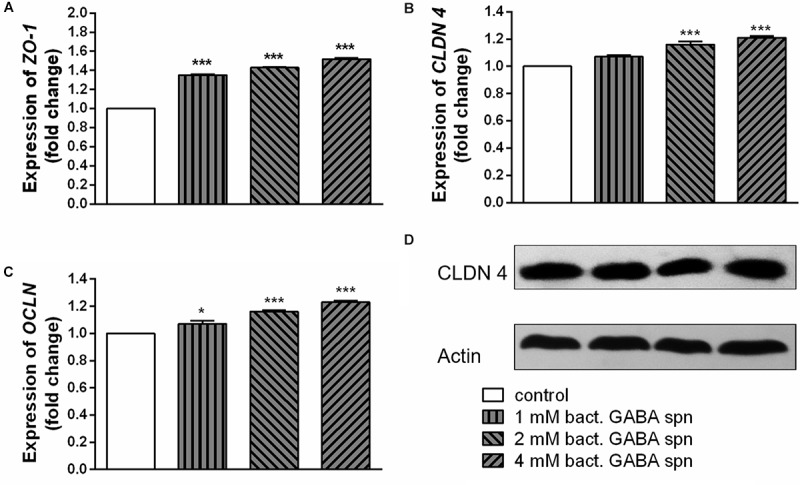
Effect of BGZLS10-17 supernatant containing bacterial GABA in three concentrations (1, 2, and 4 mM) on the expression of tight junction proteins. mRNA expression levels of *zonulin* **(A)**, *occludin* **(B),** and *claudin 4* **(C)** and representative western blot showing claudin 4 protein expression **(D)** in differentiated Caco-2 cells. The statistical significance of mRNAs expression is shown (^∗^*p* < 0.05; ^∗∗∗^*p* < 0.001); bact., bacterial.

**FIGURE 6:**
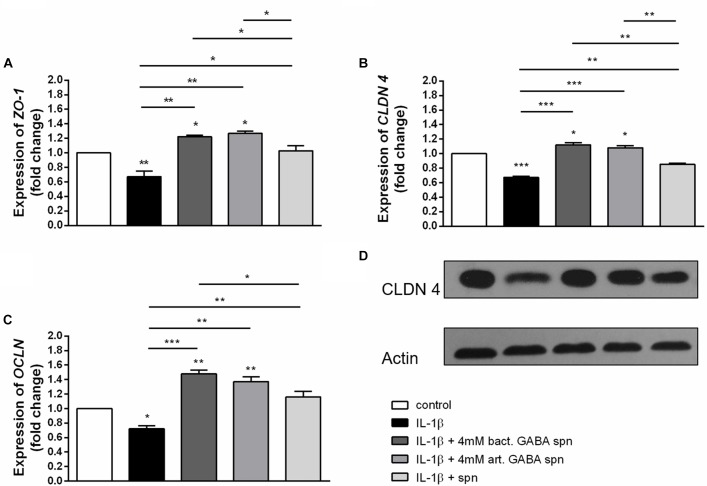
Correlation between IL-1β and protective effect of BGZLS10-17 supernatant without GABA, supernatant containing 4 mM bacterial GABA, or supernatant with 4 mM artificial GABA mRNA expression levels of *zonulin* **(A)**, *occludin* **(B)** and *claudin 4* **(C)** and representative western blot showing claudin 4 protein expression **(D)** in differentiated Caco-2 cells treated with IL-1β together with the BGZLS10-17 supernatant without GABA, supernatant containing 4 mM bacterial GABA, or supernatant with 4 mM artificial GABA; The statistical significance of mRNAs expression is shown (^∗^*p* < 0.05, ^∗∗^*p* < 0.01, ^∗∗∗^*p* < 0.001); bact., bacterial; art., artificial.

**FIGURE 7:**
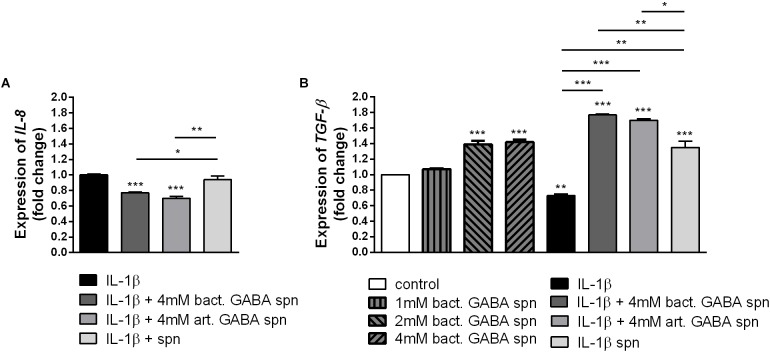
The effect of BGZLS10-17 supernatant on the cytokine production. mRNA expression levels of *IL-8* stimulated by IL-1β with and without BGZLS10-17 supernatant without GABA, 4 mM bacterial GABA in supernatant, and 4 mM artificial GABA in supernatant **(A)** and *TGF-β* expression levels in presence of BGZLS10-17 supernatant containing bacterial GABA in three concentrations (1, 2, and 4 mM) and expression of *TGF-β* in presence of IL-1β, or IL-1β together with the BGZLS10-17 supernatant containing 4 mM bacterial GABA, and supernatant with addition of 4 mM artificial GABA **(B)** in differentiated Caco-2 cells. The statistical significance of mRNAs expression is shown (^∗^*p* < 0.05; ^∗∗^*p* < 0.01, ^∗∗∗^*p* < 0.001), bact., bacterial; art., artificial.

Finally, the important probiotic feature is the maintenance of gut barrier integrity (Bron et al., 2017) and immunomodulatory ability (Živkovic et al., 2016). There are evidences that reveal the GABA signaling system is involved in maintenance of immune system homeostasis (Jin et al., 2013). IL-1β has a central role in promoting intestinal inflammation, partially by stimulating IEC s to produce IL-8, the potent neutrophil and T-lymphocyte chemoattractant (Schuerer-Maly et al., 1994). This proinflammatory cascade is implicated in different intestinal inflammatory diseases. The epithelium in inflamed intestinal segments is characterized by a reduction of tight junction strands (Schulzke et al., 2009). Inflammatory bowel disease (IBD) patients demonstrate a loss of tight junction barrier function, increased proinflammatory cytokine production, and immune dysregulation (Edelblum and Turner, 2009). Regarding this, we investigated the effects of BGZLS10-17, the best GABA-producing strain supernatant containing different (1 mM, 2 mM and 4 mM) non-toxic (data not shown) concentrations of GABA on the expression of tight junction proteins in differentiated Caco-2 cells monolayer. Interestingly, supernatants containing all three GABA concentrations significantly stimulated the expression of *ZO-1* and *OCLN* mRNA (Figure 5A,B), while supernatants containing 2 and 4 mM GABA significantly stimulated the expression of *CLDN 4* (Figure 5C,D). Ct values are presented in the Supplementary Table 1. In addition to promoting inflammatory cascade, IL-1β has been shown to induce increase in intestinal epithelial tight junction permeability, the mechanism shown to be an early event in the development of different inflammatory conditions (Al-Sadi et al., 2008). In that sense, we investigated whether these treatments can alleviate deleterious effects of IL-1β on tight junction proteins. The treatment of differentiated Caco-2 cells monolayer with IL-1β significantly reduced the expression of all tight junction mRNAs (Figure 6A–C), as well as claudin protein (Figure 6D), while addition of either of these treatments reverted significantly all these effects of IL-1β on tight junction. It is interesting that the supernatants containing GABA have more significant protective effect on tight junction proteins in comparison to supernatant without GABA. Such pronounced potential of the supernatant to protect tight junction proteins from deleterious effect of inflammation is in accordance with the recent results related to GABA potential to improve the gut barrier function, acting through selective up-regulation of Mucin-1 protein in isolated pig jejunum (Braun et al., 2015). Additionally, El-Hady et al. (2017) demonstrated that GABA administration reduced the degenerative changes in the jejunal epithelial cells and significantly improved the survival of villi and crypts in gamma-irradiated rats. Considering this we further investigated whether BGZLS10-17 supernatant with highest GABA concentration (4 mM), supernatant with addition of 4mM artificial GABA, and supernatant without GABA, may have affect on IL-1β induced IL-8 production by differentiated Caco-2 cells monolayer. The results are very promising, disclosing that treatment of Caco-2 by supernatant containing GABA produced by BGZLS10-17, as well as supernatant containing artificial GABA, significantly decreased *IL-8* production by Caco-2 induced by IL-1β in this experimental setting. Interestingly, treatment with supernatant without GABA had no modulatory effects on *IL-8* mRNA expression by Caco-2 cells (Figure 7A). TGF-β has significant role in the maintenance of epithelial barrier integrity as well as restriction of unrestrained inflammation (Planchon et al., 1994). In that sense, looking for a possible mechanism on preventing the disruption of tight junction proteins expression in IL-1β treated Caco-2 cells, we analyzed the modulatory effects of the treatments on TGF-β production by Caco-2 cells. The treatments with supernatants containing 2 and 4 mM bacterial GABA significantly stimulated the expression of *TGF-β* (Figure 7B). Importantly, all supernatants prevented the decreasement of *TGF-β* expression by Caco-2 cells induced by IL-1β (Figure 7B). Interestingly, the supernatants containing GABA (bacterial or artificial) have more significant effect on prevention of IL-1β induced decreasement in TGF-β expression by Caco-2 cells. All these results point to the potential of supernatants obtained from BGZLS10-17 culture in different media to prevent the deleterious effects of IL-1β on Caco-2 cells. Interestingly, the presence of GABA (bacterial or artificial) in supernatant have shown significant additional protective effect on IL-1β treated Caco-2 cells. Almost identical modulatory effect of supernatant containing bacterial GABA and artificial GABA in same concentration additionally supports the hypothesis that GABA is an important molecule that contributes to the protective effect of BGZLS10-17 supernatant on IL-1β induced disruption of the intestinal barrier. Additionally, these results together with the adhesive properties of BGZLS10-17 and its capability to decrease the adhesion of important gut pathogens point to the promising role of BGZLS10-17 in the treatment of chronical gut infections characterized by aggravated inflammation and disrupted epithelial integrity.

## Conclusion

The results of this study support the idea that using GABA producing BGZLS10-17 probiotic strain could be a good strategy to modulate immunological response in various inflammatory diseases, and at the same time, BGZLS10-17 stands out as a promising candidate for adjunct starter culture for production of innovative added-value GABA-enriched dairy products and offers new perspectives in designing the novel functional foods.

## Author Contributions

SSB performed main work, analyzed and interpreted the data, and drafted the work. JD conceived and designed the experiments, performed part of the experiments, analyzed and interpreted the data, and critically revised the manuscript. MD performed part of the experiments and analyzed and interpreted the data. KV performed part of the experiments and analyzed and interpreted the data. NG supervised the work, analyzed and interpreted the data, and drafted the work. SM conceived and designed the work, performed part of the experiments, and analyzed and interpreted the data. MT conceived and designed the experiments, supervised the work, analyzed and interpreted the data, and critically revised the manuscript. All authors finally approved the version to be published and agreed to be accountable for all aspects of the work in ensuring that questions related to the accuracy or integrity of any part of the work are appropriately investigated and resolved.

## Conflict of Interest Statement

The authors declare that the research was conducted in the absence of any commercial or financial relationships that could be construed as a potential conflict of interest.

## Footnotes

1 http://www.ncbi.nlm.nih.gov/BLAST
