# Supplementary material for: GABA-Producing Natural Dairy Isolate From Artisanal Zlatar Cheese Attenuates Gut Inflammation and Strengthens Gut Epithelial Barrier in vitro
Source: Front Microbiol. 2019 Mar 18;10:527. doi: 10.3389/fmicb.2019.00527 (PMC6431637; doi:10.3389/fmicb.2019.00527)
Supplement: Supplementary file 1 [file Table_1.docx]

|  |  | Ct value |  |  |  |
| --- | --- | --- | --- | --- | --- |
|  | Replicate | Actin | Zonulin (*ZO-1*) | Claudin (*CLDN4*) | Occludin (*OCLN*) |
| Control | 1 | 19.942 | 28.992 | 24.146 | 27.326 |
|  | 2 | 20.002 | 29.183 | 24.045 | 27.007 |
|  | 3 | 19.869 | 29.077 | 24.096 | 27.325 |
| Supernatant with 1 mM GABA | 1 | 18.801 | 27.821 | 23.134 | 26.178 |
|  | 2 | 19.378 | 28.687 | 23.174 | 26.042 |
|  | 3 | 19.089 | 27.893 | 23.156 | 26.425 |
| Supernatant with 2 mM GABA | 1 | 20.178 | 28.571 | 24.343 | 27.404 |
|  | 2 | 19.942 | 28.614 | 24.39 | 27.551 |
|  | 3 | 20.068 | 28.912 | 23.289 | 26.315 |
| Supernatant with 4 mM GABA | 1 | 19.723 | 27.871 | 24.432 | 26.604 |
|  | 2 | 19.605 | 27.714 | 23.895 | 26.482 |
|  | 3 | 19.658 | 28.812 | 22.299 | 26.710 |

**Supplementary Table 1.** Ct values for Figure 5 (A-C)
